# Why learn research methodology?

**DOI:** 10.4103/0301-4738.49389

**Published:** 2009

**Authors:** Barun Kumar Nayak

**Affiliations:** P. D. Hinduja National Hospital and Medical Research Centre, Veer Savarkar Marg, Mahim, Mumbai - 400 016, India. E-mail: ijo.editor@gmail.com

At the first glance, most of us would assume that this editorial is only for those who are involved in research and is certainly not for me. However, we should realize that medical science is not an exact science like physics or many other branches of science. For example we have been successful in sending unmanned space craft to the moon with precision and can accurately predict a lunar eclipse even 500 years from now. On the other hand when it comes to medicine we are dealing with human beings and no two humans are similar. The effects of our interventions are variable. It is an ever evolving science based on research. We have entered into an era of evidence-based medicine (EBM)[1] which aims to apply evidence gained by a scientific method to change current medical practice. It seeks to assess the quality of evidence relevant to the risks and benefits of treatment (including lack of treatment).[2] “EBM is the conscientious, explicit and judicious use of the current best evidence in making decisions about the care of individual patients” according to the center for EBM.[2] The basis of EBM is constant research. The aim of this editorial is to emphasize that each and every doctor needs to understand the basics of the science and art of research methodology. Conduction of research involves science and subsequent publication requires scientific writing which is an art in itself.

Why should I do research? In the era of EBM, all decisions regarding investigations, diagnosis and treatment are taken on the basis of research. We constantly strive to improve, through research, the management of a particular case. The wider cause for conducting research is to improve patient care, a great service to the mankind. The other narrower reasons could be personal satisfaction of contributing to science and the society, recognition and appreciation by peer in the profession, acquiring a job, getting a job promotion or even to retain a job. We are fast approaching towards the publish or perish principle adopted by the west.

Who should learn Research Methodology? Undergraduate medical students need to learn it for a stronger foundation for their future; postgraduate students have no choice as a thesis/dissertation is the requirement for a Masters degree. General practitioners or consultants involved in private practice cannot shy away from understanding research as they have to deal with different types of cases which may not be straight forward enough. Consultants holding teaching posts, have to guide their students for conducting their thesis. All those who hold administrative posts have to take decisions for their organizations and the research outcomes help them to a large extent in this regard. Policy makers take the help of research while framing policies. Hence, anyone who is related to medical science needs to understand research.

Why should I learn Research Methodology? From initiation of a research idea to the ultimate culmination in publication there are many segments; initiation of a research idea, thorough literature search, formulation of a research question, proper study design, possible source of funding, conduction of research, analysis of data obtained, proper interpretation of results and publication in a peer-reviewed journal. For those who are involved in conduction of research, it is mandatory that they perform the research well to curtail wastage of time and money. They are also expected to publish it later in a peer-reviewed journal so that the research can be presented to the world without any ambiguity. Those who are not involved in conducting research themselves, are the end users of the fruit of such tedious efforts and should at least be able to differentiate between good and bad science. Only good science needs to be adopted and followed. There are many examples of biased researches in literature.[3,4] It is not possible to differentiate between biased research outcomes from unbiased ones without a proper understanding of each aspect of research. Having a good foundation of research at any level may help in pursuing a research career in the future. Any treatment instituted on the basis of published evidence in a peer- reviewed journal becomes a very good defence for doctors in the court of law in cases of dispute.

When it is obvious that everyone should learn research methodology, then where is the lucuna? In the undergraduate curriculum research methodology and epidemiology is covered under preventive and social medicine. Unfortunately, not too much importance is given either by the teachers or by the students at the undergraduate level of learning. This lacuna is carried forward during the postgraduate course where a thesis / dissertation is the mandatory requirement. This is pushed in the background due to casual attitude aided by the lack of infrastructure and knowledge.[5–7] After this stage, the opportunities are few and far between most of us who are not involved in postgraduate teaching. However, it is crystal clear with the earlier discussion that research is of paramount importance for all of us without any exception.

The Indian Journal of Ophthalmology (IJO) in association with P.D. Hinduja National Hospital and Medical Research Center, Mumbai, has taken the initiative to bridge this gap by conducting comprehensive workshops on “Medical Research and Scientific Writing”. The 5^th^ workshop in the series has been slated for 30^th^ and 31^st^ May, 2009 in Mumbai. It will deal with focused literature search, conduction of proper research, art of scientific writing and critical analysis of published papers. It is recommended for medical practitioners, teachers and undergraduate as well as postgraduate students of any discipline.

The article by Parikh *et al*,[8] entitled “Likelihood ratios: Clinical application in day to day to practice” in this issue of IJO is another step towards bridging the gap. The authors need to be complemented for presenting this difficult topic in a simplified and lucid manner. “This is the best write up on likelihood ratios that I have ever read” was the comment by one of the reviewer of this article.

I wish and hope to infuse interest amongst the medical fraternity on an important and relevant but relatively neglected area that is medical research.
